# Nicorandil Prevents Gα_q_-Induced Progressive Heart Failure and Ventricular Arrhythmias in Transgenic Mice

**DOI:** 10.1371/journal.pone.0052667

**Published:** 2012-12-20

**Authors:** Masamichi Hirose, Yasuchika Takeishi, Tsutomu Nakada, Hisashi Shimojo, Toshihide Kashihara, Ayako Nishio, Satoshi Suzuki, Ulrike Mende, Kiyoshi Matsumoto, Naoko Matsushita, Eiichi Taira, Fumika Sato, Mitsuhiko Yamada

**Affiliations:** 1 Department of Molecular and Cellular Pharmacology, Iwate Medical University School of Pharmaceutical Sciences, Shiwa, Iwate, Japan; 2 Department of Cardiology and Hematology, Fukushima Medical University, Fukushima, Fukushima, Japan; 3 Department of Molecular Pharmacology, Shinshu University School of Medicine, Matsumoto, Nagano, Japan; 4 Department of Pathology, Shinshu University School of Medicine, Matsumoto, Nagano, Japan; 5 The Research Center for Human and Environmental Science, Shinshu University, Matsumoto, Nagano, Japan; 6 Cardiovascular Research Center, Division of Cardiology, Rhode Island Hospital & The Alpert Medical School of Brown University, Providence, Rhode Island, United States of America; 7 Department of Pharmacology, Iwate Medical University School of Medicine, Shiwa, Iwate, Japan; Georgia State University, United States of America

## Abstract

**Background:**

Beneficial effects of nicorandil on the treatment of hypertensive heart failure (HF) and ischemic heart disease have been suggested. However, whether nicorandil has inhibitory effects on HF and ventricular arrhythmias caused by the activation of G protein alpha q (Gα_q_) -coupled receptor (GPCR) signaling still remains unknown. We investigated these inhibitory effects of nicorandil in transgenic mice with transient cardiac expression of activated Gα_q_ (Gα_q_-TG).

**Methodology/Principal Findings:**

Nicorandil (6 mg/kg/day) or vehicle was chronically administered to Gα_q_-TG from 8 to 32 weeks of age, and all experiments were performed in mice at the age of 32 weeks. Chronic nicorandil administration prevented the severe reduction of left ventricular fractional shortening and inhibited ventricular interstitial fibrosis in Gα_q_-TG. SUR-2B and SERCA2 gene expression was decreased in vehicle-treated Gα_q_-TG but not in nicorandil-treated Gα_q_-TG. eNOS gene expression was also increased in nicorandil-treated Gα_q_-TG compared with vehicle-treated Gα_q_-TG. Electrocardiogram demonstrated that premature ventricular contraction (PVC) was frequently (more than 20 beats/min) observed in 7 of 10 vehicle-treated Gα_q_-TG but in none of 10 nicorandil-treated Gα_q_-TG. The QT interval was significantly shorter in nicorandil-treated Gα_q_-TG than vehicle-treated Gα_q_-TG. Acute nicorandil administration shortened ventricular monophasic action potential duration and reduced the number of PVCs in Langendorff-perfused Gα_q_-TG mouse hearts. Moreover, HMR1098, a blocker of cardiac sarcolemmal K_ATP_ channels, significantly attenuated the shortening of MAP duration induced by nicorandil in the Gα_q_-TG heart.

**Conclusions/Significance:**

These findings suggest that nicorandil can prevent the development of HF and ventricular arrhythmia caused by the activation of GPCR signaling through the shortening of the QT interval, action potential duration, the normalization of SERCA2 gene expression. Nicorandil may also improve the impaired coronary circulation during HF.

## Introduction

It is well known that the abnormalities in coronary hemodynamics in systolic heart failure (HF) are frequent. Myocardial oxygen demand and consumption are increased and myocardial perfusion is also impaired in HF, which can result in myocardial ischemia, necrosis and apoptosis. This is potentially a factor contributing to progressive heart failure. Nicorandil is an ATP-sensitive K^+^ (K_ATP_) channel opener and a nitric oxide donor, which dilates epicardial and resistance coronary arteries as well as peripheral resistance arterioles and systemic veins. Thus, nicorandil increases coronary blood flow, reduces preload and afterload, and exerts an antianginal action [1], [2]. In addition, beneficial hemodynamic effects of nicorandil have also been demonstrated in acute HF, suggesting a possible effect of this drug in the treatment of HF [3]. In fact, intravenous administration of nicorandil attenuated exercise-induced LV diastolic dysfunction in individuals with hypertrophic cardiomyopathy, probably as a result of its beneficial effect on abnormal coronary microcirculation [4]. Moreover, chronic nicorandil administration prevented the development of HF in Dahl salt-sensitive hypertensive rats as a result of the promotion of myocardial capillary and arteriolar growth [5]. These findings support the notion that nicorandil may ameliorate HF associated with defective coronary microcirculation. Our previous study demonstrated a direct effect of nicorandil on ventricular myocardium (i.e. shortening of ventricular action potential), leading to the prevention of ventricular tachyarrhythmias during myocardial ischemia [6].

It is known that the G protein αq-coupled receptor (GPCR) signaling pathway plays a critical role in the development of cardiac hypertrophy and HF [7]–[9]. Our previous study demonstrated that a transgenic mouse with transient cardiac expression of activated Gα_q_ (Gα_q_-TG) developed chronic HF and ventricular tachyarrhythmias [10]–[12]. While nicorandil may prove beneficial for the treatment of hypertensive heart failure as well as of ischemic heart disease, it remains unknown whether nicorandil has inhibitory effects on the development of HF and ventricular arrhythmias caused by activation of the G_q_ signaling pathway. We hypothesized that nicorandil can prevent the development of HF and HF-induced ventricular arrhythmias through improvement of coronary hemodynamics and ventricular electrophysiological property. In the present study, we investigated the inhibitory effects of nicorandil on HF and ventricular arrhythmias in Gα_q_-TG mice.

## Materials and Methods

The experimental protocol was approved by the institutional animal experiments committee and complied with the Guide for Care and Use of Laboratory Animals published by the US National Institutes of Health (NIH publication 85-23, revised 1996). The animal experiments were also approved by the Shinshu University School of Medicine Animal Studies Committee (approval ID 200044).

### Experimental Animals

A transgenic mouse (Gα_q_-TG mouse) with transient cardiac expression of activated Gα_q_ was used [12]. The wild-type (WT) mice used in this study are littermates from the non-transgenic mice. The genotypes of the WT and Gα_q_-TG mice were identified by polymerase chain reaction (PCR) with the use of tail genomic DNA as a template as previously reported. Our previous studies demonstrated that Gα_q_-TG mice developed HF but not ventricular arrhythmias at the age of 16 weeks, whereas they developed both by 32 weeks. Therefore, to examine effects of chronic nicorandil administration on HF and ventricular arrhythmias, nicorandil (6 mg/kg/day) or vehicle was orally administered to Gα_q_-TG mice from 8 to 32 weeks of age. In addition, to examine potential basal effects of long-term nicorandil treatment in WT mice, nicorandil (6 mg/kg/day) or vehicle was also administered to WT mice from 8 to 32 weeks of age. All experiments were performed in 32-week-old mice. All mice were anesthetized with sodium pentobarbital (30 mg/kg) applied intraperitoneally. The adequacy of anesthesia was monitored by watching heart rate and the frequency and the degree of motion of the sternum and of movement of the extremities.

### Echocardiography

WT, nicorandil-treated WT, vehicle-treated Gα_q_-TG, and nicorandil-treated Gα_q_-TG female mice (n = 6 each) were anesthetized, and cardiac function was assessed with echocardiography (GE Yokogawa Medical System, Tokyo, Japan) as previously described [13]. Hearts were viewed at the level of the papillary muscles along the short axis. In M-mode tracings, the average of three consecutive beats was used to measure the following parameters: interventricular septum thickness, left ventricular end-diastolic dimension (LVEDd), end-systolic dimension (LVESd) and fractional shortening (LVFS), which was calculated as follows: (LVEDd - LVESd)/LVEDd ×100%. Echocardiography was also performed in WT and Gα_q_-TG mice at the age of 8 weeks before treatment with nicorandil.

### Gross Anatomy and Histology

WT, nicorandil-treated WT, vehicle-treated Gαq-TG, and nicorandil-treated Gαq-TG female mice (n = 10 each) were anesthetized and treated with sodium heparin (500 USP units/kg i.v.). After a midline sternal incision, hearts were quickly excised. The hearts were fixed with a 30% solution of formalin in phosphate-buffered saline at room temperature for more than 24 hours, embedded in paraffin, and then cut serially from the apex to the base. Six sections were stained with hematoxylin/eosin or Masson’s trichrome for histopathological analysis. Transverse sections were captured digitally, and the cross-sectional diameter of at least 20 cardiomyocytes in each section was measured using the image analyzing software MacSCOPE (MITANI Corporation, Tokyo) on a Macintosh computer. The measurements were performed on 3 sections in each preparation and averaged. To assess the degree of fibrosis, digital microscopic images were taken from the sections stained with Masson’s trichrome stain using light microscopy with a digital camera system. The measurements were performed on 3 images from different parts of the left ventricle in each preparation as described previously [14]. The fibrosis fraction was obtained by calculating the ratio of total connective area to total myocardial area from 3 images in each preparation.

### Western Blot Analysis

Total protein was prepared from the ventricular myocardium of anesthetized WT, vehicle-treated Gα_q_-TG and nicorandil-treated Gα_q_-TG mice (n = 6 each) using a lysis buffer (Cell Signaling Technology, Inc. Danvers, MA) to examine the protein expression of TRPC isoforms. Protein concentrations assayed, and equal amounts of the proteins were subjected to 10% SDS-PAGE and transferred to PVDF membranes. To ensure equivalent protein loading and to verify efficient protein transfer, membranes were stained with Ponceau S before incubating with primary isoform-specific antibodies against TRPC isoforms (TRPC 3 and 6; SIGMA, Saint Louis, MO) and actin [15]. Immunoreactive bands were detected with an ECL kit (Amersham Biosciences Corp., Piscataway, NJ). The densitometric intensity of bands representing TRPC isoforms was normalized to that of actin. In addition, to examine potential basal effects of long-term nicorandil treatment in WT mice, a western blot analysis was performed in vehicle-treated and nicorandil-treated WT mice (n = 5 each).

### Quantification of mRNA by Real-time PCR

Total RNA was prepared from the ventricular myocardium of anesthetized WT, vehicle-treated Gα_q_-TG and nicorandil-treated Gα_q_-TG mice (n = 6 each) with Isogen (Nippon Gene Co. LTD., Tokyo, Japan) according to the manufacturer’s instructions. One microgram of total RNA was used as a template for reverse transcription with the SuperScript First-Strand synthesis system for qRT-PCR (Invitrogen, Carlsbad, CA). To generate a standard curve for mRNA quantification, partial cDNA fragments of atrial natriuretic factor (ANF), B-type natriuretic peptide (BNP), β-myosin heavy chain (β-MHC), Kir6.1, Kir6.2, sulfonylurea receptor (SUR) 1, SUR2A, SUR2B, endothelial nitric oxide synthese (eNOS), inducible NOS (iNOS), connective tissue growth factor (CTGF), collagen type 1, phspholamban (PLB), sarco(endo)-plasmic reticulum Ca^2+^-ATPase 2 (SERCA2), sodium/calcium exchanger 1 (NCX1) and acidic ribosomal protein P0 (ARPP0) were amplified from the heart cDNA by PCR with DNA polymerase Ex Taq HS (Takara Bio, Shiga, Japan) and subcloned into the pGEM-T Easy vector (Promega, Madison, WI). Real-time PCR was performed with an ABI Prism 7900HT Sequence Detection System (Applied Biosystems, Foster City, CA). The PCR mixture (20 µl) contained FastStart Universal SYBR Green Master (Rox) (Roche Diagnostics), standard cDNA (10^2^ to 10^6^ ng per reaction) or 0.2 µl of reverse-transcribed cDNA samples and 100 nM of forward and reverse primers. All primers used are listed in Table S1. To choose a suitable internal control for this study, glyceraldehyde 3-phosphate dehydrogenase, β-actin, β-glucuronidase, and ARPP0 were tested, and it was found that the expression of ARPP0 mRNA was most constant between the groups. Thus, the expression of each gene was normalized to that of ARPP0 mRNA. The specificity of the method was confirmed by a dissociation analysis according to the instructions supplied by Applied Biosystems. In addition, to examine potential basal effects of long-term nicorandil treatment in WT mice, the real-time PCR was performed in WT and nicorandil-treated WT mice (n = 5 each).

### Electrocardiography (ECG)

WT, nicorandil-treated WT, vehicle-treated Gα_q_-TG, and nicorandil-treated Gα_q_-TG female mice (n = 10 each) were anesthetized. Electrocardiography (ECG) lead II was recorded for 10 min in all mice. ECG lead II was recorded and filtered (0.1 to 300 Hz), digitized with 12-bit precision at a sampling rate of 1000 Hz per channel (Microstar Laboratories Inc., Bellevue, WA, USA), transmitted into a microcomputor and saved on CD-ROM.

### Monophasic Action Potential (MAP)

WT and vehicle-treated Gα_q_-TG mice were anesthetized and treated with sodium heparin (500 USP units/kg i.v.). After a midline sternal incision, hearts were quickly excised and connected to a modified Langendorff apparatus. A polyterafluoroethylene-coated silver unipolar electrode was used to stimulate the epicardial surface of the anterior left ventricle at twice the diastolic threshold current with a duration of 1 ms. A monophasic action potential (MAP) electrode was placed on the epicardial surface of the posterior left ventricle, and MAP was recorded for 10 sec at a basic cycle length of 200 ms to measure MAP duration. Each preparation was perfused under constant flow conditions with oxygenated (95% oxygen, 5% CO_2_) Tyrode’s solution containing in mM: NaCl, 141.0; KCl, 5.0; CaCl_2_, 1.8; NaHCO_3_, 25.0; MgSO_4_, 1.0; NaH_2_PO_4_, 1.2; HEPES, 5; and dextrose, 5.0 (pH of 7.4 at 36±1°C). Perfusion pressure was measured with a pressure transducer (Nihon Kohden Co, Tokyo, Japan) and maintained within a pressure range (50–60 mmHg) by adjusting flow. The MAP signals were filtered (0.3 to 300 Hz), amplified (1000×) and recorded. Perfusion pressure and flow were continuously monitored during each experiment.

First, to examine direct effects of nicorandil on ventricular action potential in Gα_q_-TG mouse hearts, MAP was recorded from the epicardial surface of the posterior left ventricle in vehicle-treated WT and Gα_q_-TG mouse hearts (n = 8 each) before and after the application of nicorandil (1 and 10 µM). Next, to examine the mechanisms underlying the effect of nicorandil on ventricular action potential, MAP was recorded in vehicle-treated Gα_q_-TG mouse hearts (n = 4) in the presence of nicorandil alone, and in the presence of nicorandil plus HMR1098 (30 µM), a blocker of cardiac sarcolemmal K_ATP_ channels.

### Electrophysiological Measurement

In all mice examined, P, PR, QRS complex, QT, and RR intervals were measured from ECG lead II. The number of premature ventricular contractions (PVCs) per minute was calculated from ECG lead II. A high incidence of PVCs (High PVC) was defined as more than 20 beats/min. In MAP signals of all Langendorff hearts, automated algorithms were used to determine depolarization time relative to a single fiducial point (i.e., the stimulus). Depolarization time was defined as the point of maximal positive derivative in the action potential upstroke (dV/dt_max_). Repolarization time was defined as the time when repolarization reached a level of 50%. MAP duration was defined as the difference between repolarization time and depolarization time.

### Data Analysis

All data are shown as the mean ± SE. An analysis of variance with Bonferroni's test was used for the statistical analysis of multiple comparisons of data. Fisher’s exact test was used to compare the incidence of VT between different conditions. P<0.05 was considered statistically significant.

### Drug

Nicorandil was kindly provided by Chugai Pharmaceutical Co. (Tokyo, Japan).

## Results

### Nicorandil Prevented the Progression of Cardiomegaly and Contractile Dysfunction in Gα_q_-TG Mice

**Figure 1:**
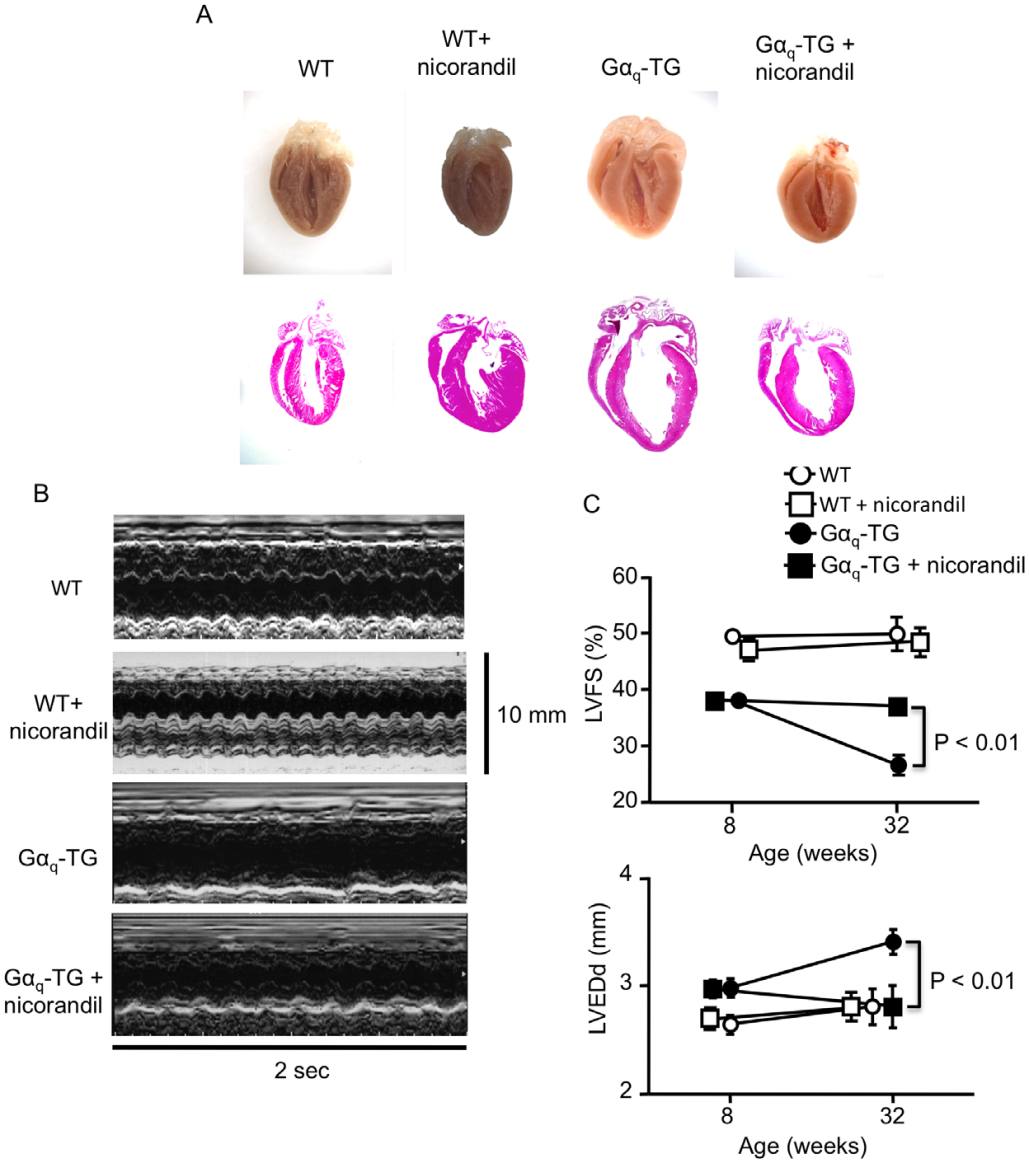
Effects of nicorandil on cardiac morphology and on the left ventricular contractile function. Panel A: Gross examination of the four-chamber section of a heart and its histology stained with hematoxylin/eosin in a WT (wild-type), WT+nicorandil, Gα_q_-TG, and Gα_q_TG+nicorandil mouse heart. Original magnification: 1.25×. Panel B: Representative M-mode echocardiograms of a WT, WT+nicorandil, Gα_q_-TG, and Gα_q_TG+nicorandil mouse at the age of 32 weeks. Panel C: Age-dependent changes in left ventricular fractional shortening (LVFS) and left ventricular end-diastolic dimension (LVEDd) in WT, WT+nicorandil, Gα_q_-TG, and Gα_q_TG+nicorandil mice. WT+nicorandil, nicorandil-treated WT; Gα_q_-TG, vehicle-treated Gα_q_-TG; Gα_q_TG+nicorandil, nicorandil-treated Gα_q_TG. Mice at age of the 32 weeks were used.

To evaluate whether chronic administration of nicorandil could prevent the progression of HF in Gα_q_-TG mice, cardiac morphology was examined in WT, nicorandil-treated WT, vehicle-treated Gα_q_-TG and nicorandil-treated Gα_q_-TG mice at the age of 32 weeks. Gross examination of the four-chamber section of the heart revealed all chambers to be dilated in the vehicle-treated Gα_q_-TG heart compared with WT and nicorandil-treated Gαq-TG hearts (Fig. 1A). The vehicle-treated Gα_q_-TG mouse exhibited marked cardiomegaly. The heart/body weight ratio increased in vehicle-treated Gα_q_-TG mice compared with WT mice, but nicorandil significantly reduced the ratio in Gα_q_-TG mice (Table 1). The left atrial size**/**tibial length ratio was also increased in vehicle-treated Gα_q_-TG compared with WT hearts. Nicorandil decreased the ratio in Gα_q_-TG hearts (Table 1). Echocardiography was performed, and representative M-mode echocardiograms are shown in Figure 1B. Compared with the WT mice, vehicle-treated Gα_q_-TG mice exhibited impaired left ventricular contractility and chamber dilation as demonstrated by the markedly reduced LVFS and the increased LVEDd (Fig. 1B and Table 2). However echocardiographic parameters such as reduced LVFS and increased LVEDd were significantly improved in nicorandil-treated Gα_q_-TG compared with vehicle-treated Gα_q_-TG mice (Fig. 1B and Table 2). Moreover, LVEDd and LVFS age-dependently changed in Gα_q_-TG mice, whereas the values in nicorandil-treated Gα_q_-TG mice at 32 weeks were similar to those at 8 weeks, indicating that nicorandil prevented the progression of cardiomegaly and contractile dysfunction in Gα_q_-TG mice (Fig. 1C).

**Table 1:** General parameters and the incidence of premature ventricular contraction (PVC) in WT, WT+nicorandil, Gα_q_-TG, and Gα_q_-TG+nicorandil mice.

Parameters	WT	WT+nicorandil	Gα_q_-TG	Gα_q_-TG+nicorandil
BW (g)	29.8±1.5	24.2±0.3^b^	29.8±1.4	29.7±1.2
HW (mg)	130±2.4	125±4.0	170±8.8^b^	148±9.5^+^
HW/BW (mg/g)	4.4±0.2	5.1±0.2	5.8±3^a^	5.0±0.5
LA/TL (mm/mm)	0.14±0.01	0.17±0.01	0.27±0.02^b^	0.21±0.02^b,+^
PVC	1/10	0/10	9/10^b^	3/10^+^
PVC (>20 beats/min)	0/10	0/10	7/10^b^	0/10^$^

Data are the mean ± SE obtained from 10 mice for each group. ^a^p<0.05, ^b^p<0.01 vs. WT, +p<0.05, ^$^p<0.01 vs. values in corresponding parameters of Gα_q_-TG.

**Table 2:** Echocardiographic parameters in WT, WT+nicorandil, Gα_q_-TG, and Gα_q_-TG+nicorandil mice.

Parameters	WT	WT+nicorandil	Gα_q_-TG	Gα_q_-TG+nicorandil
IVS (mm)	0.76±0.05	0.68±0.03	0.55±0.04^b^	0.62±0.07^a^
LVEDd (mm)	2.8±0.12	2.8±0.07	3.4±0.12^ b^	2.8±0.16^$^
LVFS (%)	49.8±2.9	47.0±1.4	26.7±1.6	37.0±0.9^b, $^

Data are the mean ± SE obtained from 6 mice for each group. ^a^p<0.05, ^b^p<0.01 vs. WT, +p<0.05, ^$^p<0.01 vs. values in corresponding parameters of Gα_q_-TG. LVEDd, left ventricular end-diastolic dimension; IVS, intraventricular septum.

### Nicorandil Inhibited Myocardial Fibrosis but not the mRNA Expression of Profibrotic Genes and Cardiomyocyte Hypertrophy in Gα_q_-TG Mice

**Figure 2:**
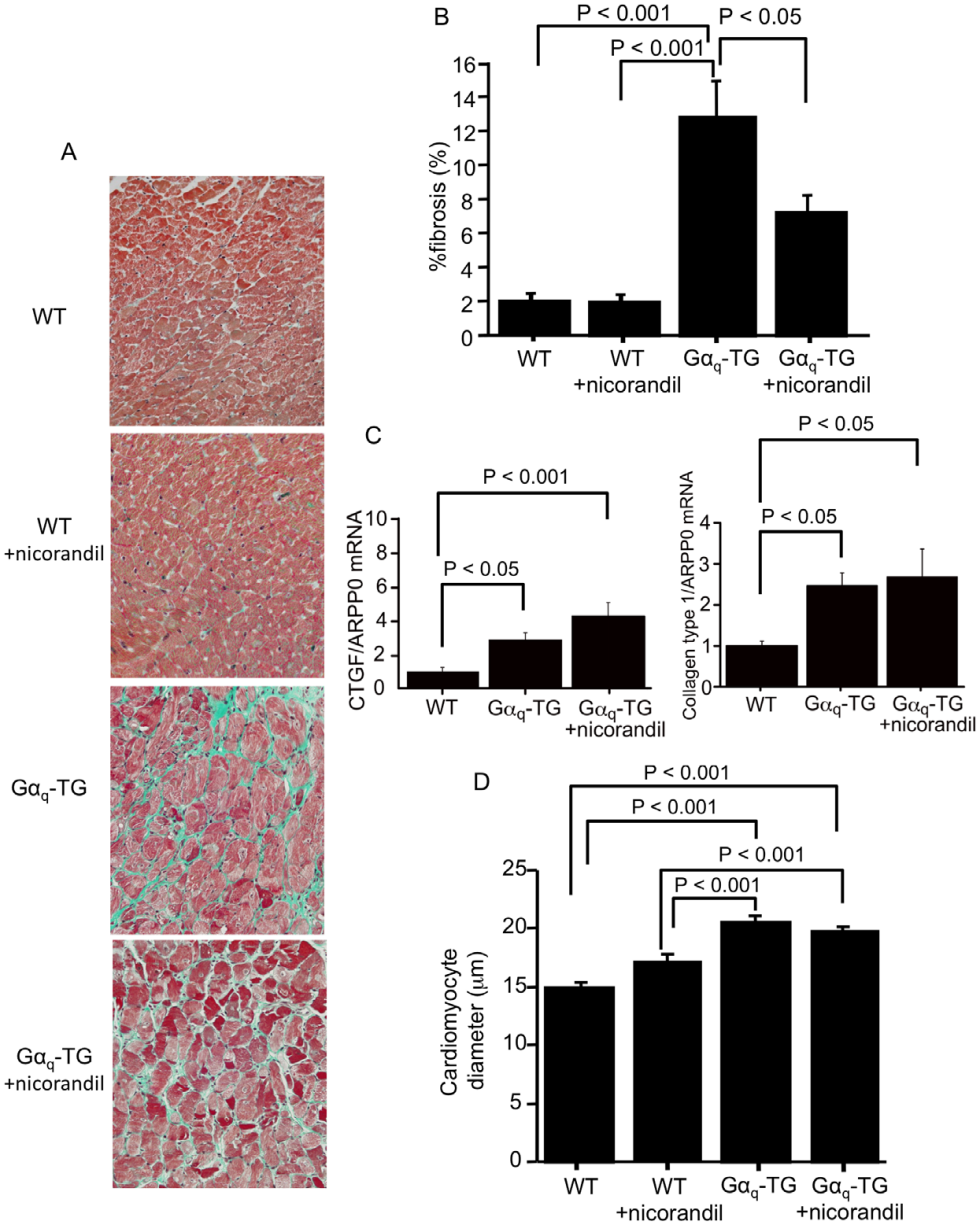
Effects of nicorandil on the left ventricular fibrosis and on connective tissue growth factor (CTGF) and collagen type 1 gene expression. Panel A: Histology of the left ventricle stained with Masson’s trichrome in a WT, WT+nicorandil, Gα_q_-TG, and Gα_q_TG+nicorandil mouse. Original magnification: 40×. Panel B: Comparison of the fibrosis fraction in the left ventricle in WT, WT+nicorandil, Gα_q_-TG, and Gα_q_TG+nicorandil mice. Panel C: Quantitative analyses of CTGF and collagen type 1 gene expression by real-time real-time reverse transcriptase-polymerase chain reaction (RT-PCR) in WT, Gα_q_-TG and Gα_q_-TG+nicorandil hearts. Data for CTGF and collagen type 1 were normalized to those for ARPP0. Data are the mean ± SE obtained from 6 mice for each group. Panel D: Comparison of cardiomyocyte size in the left ventricle in WT, WT+nicorandil, Gα_q_-TG, and Gα_q_TG+nicorandil mice.

We examined the effects of chronic nicorandil administration on left ventricular myocardial fibrosis and cardiomyocyte hypertrophy in WT, vehicle-treated Gα_q_-TG and nicorandil-treated Gα_q_-TG mice at the age of 32 weeks. Extensive interstitial fibrosis in the left ventricle was observed in vehicle-treated Gα_q_-TG hearts compared with WT and nicorandil-treated Gα_q_-TG hearts (Fig. 2A). The degree of myocardial fibrosis in the left ventricle was significantly greater in vehicle-treated Gα_q_-TG mice than in WT mice (Fig. 2B). Nicorandil significantly reduced the increased interstitial fibrosis in the left ventricle of Gα_q_-TG mice (Fig. 2B). We next examined the expression of profibrotic genes, such as the genes for CTGF and collagen type 1, to investigate whether these morphological observations were accompanied by alterations in gene expression relevant to fibrotic changes. The expression of CTGF and collagen type 1 was significantly upregulated in Gα_q_-TG hearts compared with WT mouse hearts (Fig. 2C). In nicorandil-treated Gα_q_-TG hearts, the gene expression of CTGF and collagen type 1 was similar to that in vehicle-treated Gα_q_-TG hearts (Fig. 2C).

Microscopic observation revealed that the cross-sectional diameter of cardiomyocytes was profoundly increased in vehicle-treated Gα_q_-TG mice compared with WT mice (Fig. 2D). The increase cross-sectional diameter was not attenuated in nicorandil-treated Gα_q_-TG mice (Fig. 2D).

### Nicorandil did not Inhibit Fetal Gene Expression and TRPC Channel Protein Levels in Gα_q_-TG Mice

**Figure 3:**
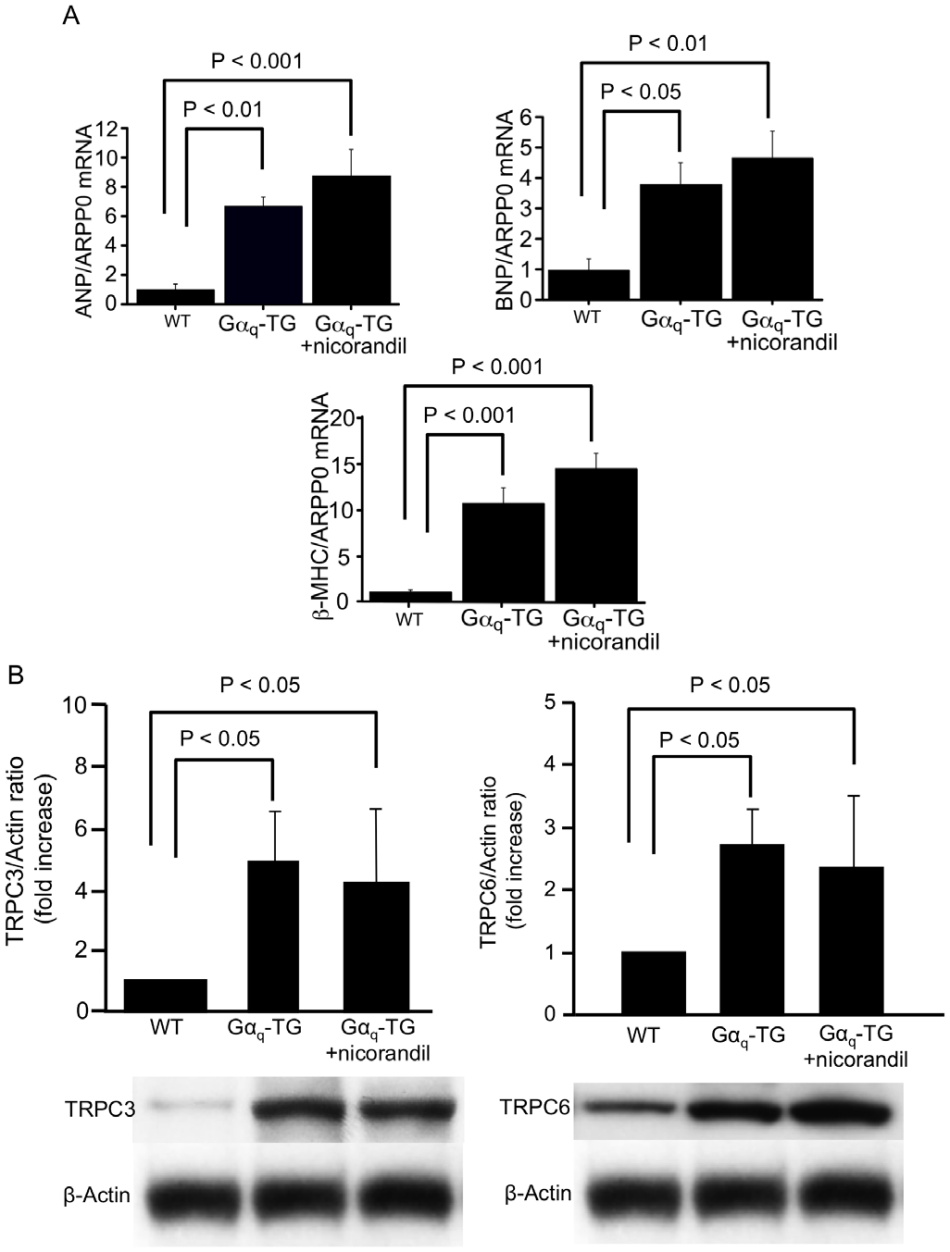
Effects of nicorandil on ANP, BNP, and β-MHC gene expressions and on protein expression of canonical transient receptor potential (TRPC) channel isoforms. Panel A: Quantitative analyses of ANP, BNP, and β-MHC gene expression by real-time RT-PCR in WT, Gα_q_-TG and Gα_q_-TG+nicorandil hearts. Data for ANP, BNP, and β-MHC were normalized to those for ARPP0. Data are the mean ± SE obtained from 6 mice for each group. Panel B: Expression of TRPC channel isoforms in WT, Gα_q_-TG and Gα_q_-TG+nicorandil hearts. TRPC isoform expression was normalized to actin expression and expressed relative to wt (set at 1). Data are the mean ± SE obtained from 6 mice for each group. ANF, atrial natriuretic factor; BNP, B-type natriuretic peptide; β-MHC, β-myosin heavy chain; ARPP0, acidic ribosomal protein P0. Mice at the age of 32 weeks were used.

We next examined the mRNA expression of fetal type genes such as ANF, β-MHC and BNP in WT, vehicle-treated Gα_q_-TG and nicorandil-treated Gα_q_-TG mice at the age of 32 weeks. Expression of ANF, BNP and β-MHC was significantly upregulated in Gα_q_-TG hearts compared with WT mouse hearts (Fig. 3A). In nicorandil-treated Gα_q_ -TG hearts, the gene expression of ANF, BNP, and β-MHC was similar to that in vehicle-treated Gα_q_-TG hearts (Fig. 3A). A recent study suggested that the activation of TRPC channels participated in the generation of cardiac hypertrophy [16]. Moreover, the protein levels of TRPC3 and 6 were increased in Gα_q_-TG mouse hearts [11]. Therefore, we examined effects of nicorandil on the protein expression of TRPC channel subtypes in Gα_q_-TG hearts. The levels of TRPC 3 and 6 channels were significantly increased in Gα_q_-TG hearts compared with WT hearts (Fig. 3B). The expression of TRPC 3 and 6 was not significantly different between vehicle-treated Gα_q_-TG and nicorandil-treated Gα_q_-TG mouse hearts (Fig. 3B).

### Gene Expression of ATP-sensitive K^+^ (K_ATP_) Channel Subunit, Nitric Oxide Synthase (NOS), and Ca^2+^-handling Proteins

**Figure 4:**
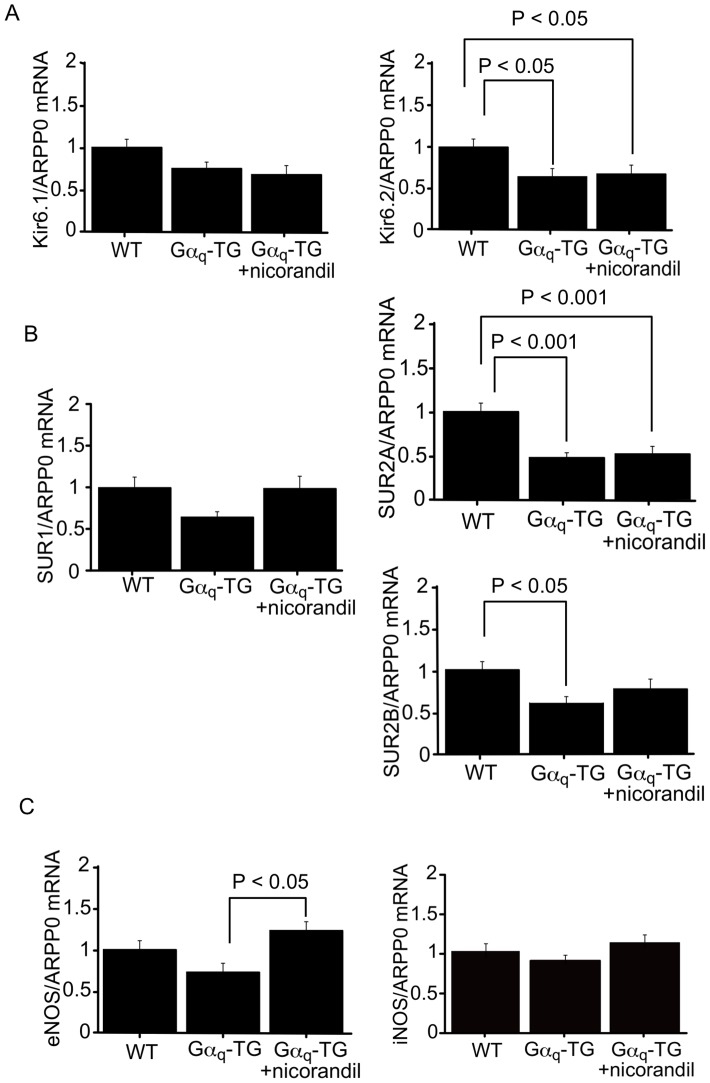
Quantitative analyses of Kir6.1 (A), Kir6.2 (A), SUR1 (B), SUR2A (B), SUR2B (B), eNOS (C), and iNOS (C) gene expression by real-time RT-PCR in WT, Gα_q_-TG and Gα_q_-TG+nicorandil hearts. Data for Kir6.1, Kir6.2, SUR1, SUR2A, SUR2B, eNOS, and iNOS were normalized to those for ARPP0. Data are the mean ± SE obtained from 6 mice for each group. SUR1, sulfonylurea receptor 1; SUR2A, sulfonylurea receptor 2A; SUR2B, sulfonylurea receptor 2B; eNOS, endothelial nitric oxide synthase; iNOS, inducible nitric oxide synthase; ARPP0, acidic ribosomal protein P0. Mice at the age of 32 weeks were used.

**Figure 5:**
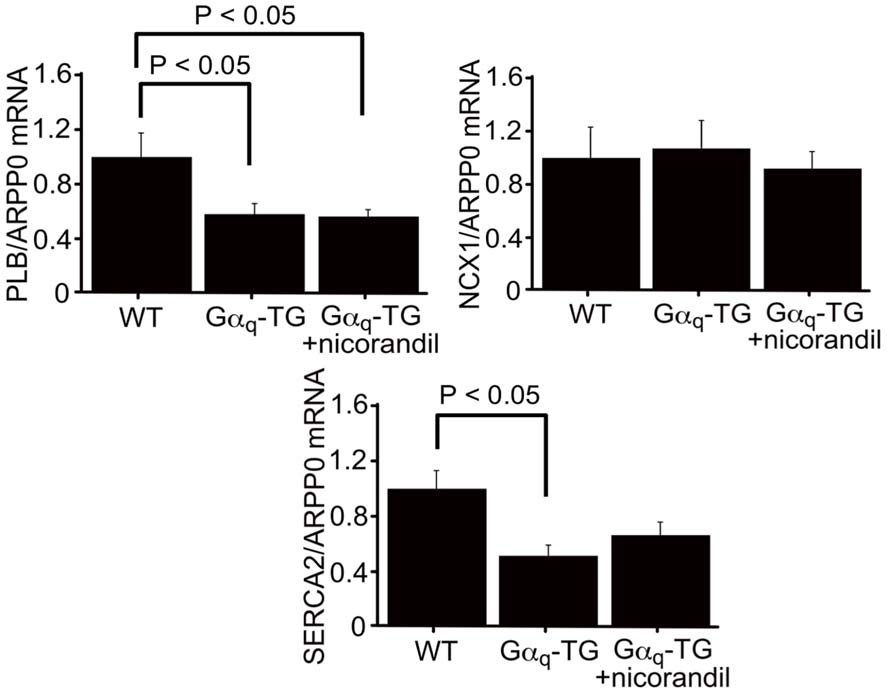
Quantitative analyses of PLB, SERCA2, and NCX1 gene expression by real-time RT-PCR in WT, Gα_q_-TG and Gα_q_-TG+nicorandil hearts. Data for PLB, SERCA2, and NCX1 were normalized to those for ARPP0. Data are the mean ± SE obtained from 6 mice for each group. PLB, phospholamban; NCX1, sodium/calcium exchanger 1; ARPP0, acidic ribosomal protein P0. Mice at the age of 32 weeks were used.

We examined the mRNA expression of ATP-sensitive K^+^ (K-ATP) channel subunits such as Kir 6.1, Kir 6.2, SUR1, SUR2A and SUR2B in WT, vehicle-treated Gα_q_-TG and nicorandil-treated Gα_q_-TG mice at the age of 32 weeks. The expression of Kir 6.2, SUR2A, and SUR2B was significantly downregulated in vehicle-treated Gα_q_-TG mouse hearts compared with that in WT mouse hearts (Fig. 4). Interestingly in nicorandil-treated Gα_q_-TG hearts, the gene expression of SUR2B but not Kir 6.2 or SUR2A was similar to that in WT hearts (Fig. 4). Previous study has shown that nicorandil upregulates eNOS expression in rat hearts with myocardial infarction [17]. We also examined the mRNA expression of eNOS and iNOS in WT, vehicle-treated Gα_q_-TG and nicorandil-treated Gα_q_-TG mice at 32 weeks of age. The mRNA expression of eNOS was significantly increased in nicorandil-treated Gα_q_-TG hearts compared with vehicle-treated Gα_q_-TG hearts (Fig. 4). Next, we examined the mRNA expression of PLB, SERCA2, and NCX1 in WT, vehicle-treated Gα_q_-TG and nicorandil-treated Gα_q_-TG mouse hearts at the age of 32 weeks. The expression of PLB and SERCA2 was significantly downregulated in vehicle-treated Gα_q_-TG hearts compared with that in WT mouse hearts (Fig. 5). Interestingly in nicorandil-treated Gα_q_-TG hearts, the gene expression of SERCA2 but not PLB was similar to that in WT hearts (Fig. 5). In contrast to the expression of PLB and SERCA2, NCX1 expression was similar among WT, vehicle-treated Gα_q_-TG and nicorandil-treated Gα_q_-TG mouse hearts.

### Nicorandil Reduces the Number of Premature Ventricular Contractions (PVCs) in Gα_q_-TG Mice

**Figure 6:**
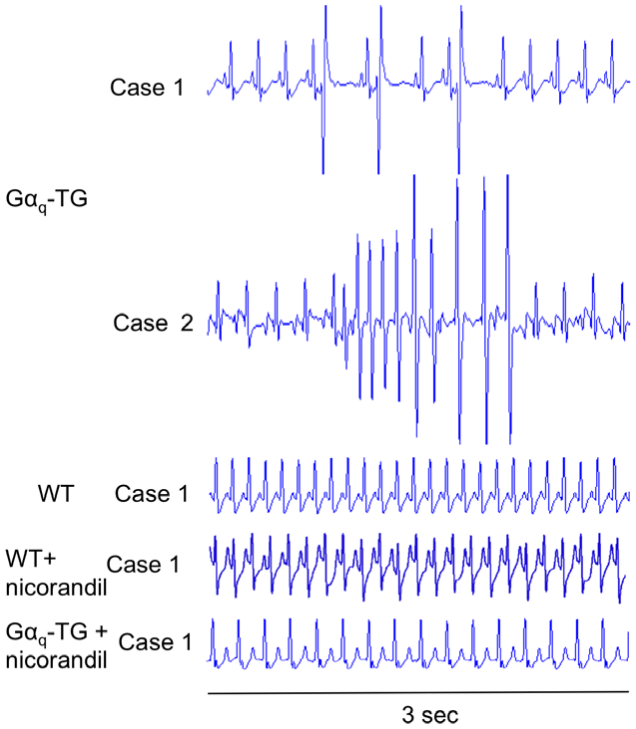
ECG lead II recordings from WT, WT+nicorandil, Gα_q_-TG, and Gα_q_+nicorandil mice. The upper 2 ECGs show premature ventricular contraction (PVC) and ventricular repetitive beats in anesthetized Gα_q_-TG mice. The lower 3 ECGs recorded from a WT, WT+nicorandil, and Gα_q_-TG+nicorandil mouse show P wave and QRS complex with a regular RR interval without any arrhythmia, indicating sinus rhythm. Mice at the age of 32 weeks were used. See text for details.

ECG lead II was recorded for 10 min in anesthetized WT, vehicle-treated Gα_q_-TG and nicorandil-treated Gα_q_-TG mice. Shown in Figure 6 are representative ECGs. The upper 2 cases show ventricular arrhythmias recorded from vehicle-treated Gα_q_-TG mice: in case 1, PVC was frequently observed; in case 2, the ECG demonstrated consecutive ventricular beats, which are features of VT. In contrast, the lower 3 cases recorded from a WT, nicorandil-treated WT, Gα_q_-TG, and nicorandil-treated Gα_q_-TG mouse showed P waves and QRS complexes with regular RR intervals without any arrhythmia, indicating a sinus rhythm. Table 1 shows the overall data for ventricular arrhythmias. Ventricular arrhythmias such as a high PVC count (more than 20 beats/min) were not observed in WT and nicorandil-treated WT mice. In contrast, a high number of PVCs was observed in 7 of 10 vehicle-treated Gα_q_-TG mice (Table 1). Interestingly, a high PVC count was not observed in any nicorandil-treated Gα_q_-TG mice tested, indicating a significant reduction of ventricular arrhythmias in nicorandil-treated Gα_q_-TG mice compared with vehicle-treated Gα_q_-TG mice.

### Nicorandil Restores the Baseline Values of P and QT Intervals in Gα_q_-TG Mice

Table 3 shows overall data for the electrophysiological parameters in WT, nicorandil-treated WT, vehicle-treated Gα_q_-TG and nicorandil-treated Gα_q_-TG mice at 32 weeks of age. All ECG parameters were longer in vehicle-treated Gα_q_-TG mice than WT mice. Interestingly, while the PR and RR interval was still prolonged in nicorandil-treated Gα_q_-TG mice compared with WT mice, the P interval and QT interval were restored to the normal level in nicorandil-treated Gα_q_-TG compared with vehicle-treated Gα_q_-TG mice.

**Table 3:** Electrocardiographic parameters in WT, WT+nicorandil, Gα_q_-TG, and Gα_q_-TG+nicorandil mice.

Parameters	WT	WT+nicorandil	Gα_q_-TG	Gα_q_-TG+nicorandil
P (msec)	18±1	16±1	25±1^b^	22±1^+^
RR (msec)	142±5	166±10	186±9^ b^	193±16^ b^
PR (msec)	39±3	43±2	62±3^ b^	55±5^ b^
QRS (msec)	16±0.5	15±0.5	19±1^a^	17±2
QT (msec)	34±2	32±2	43±2^b^	34±3^+^

Data are the mean ± SE obtained from 6 mice for each group. ^a^p<0.05, ^b^p<0.01 vs. WT, +p<0.05 vs. values in corresponding parameters of vehicle-treated Gα_q_-TG.

### Acute Application of Nicorandil Shortens the Prolonged Left Ventricular MAP Duration and Prevents Ventricular Tachyarrhythmias in Gα_q_-TG Mice

**Figure 7:**
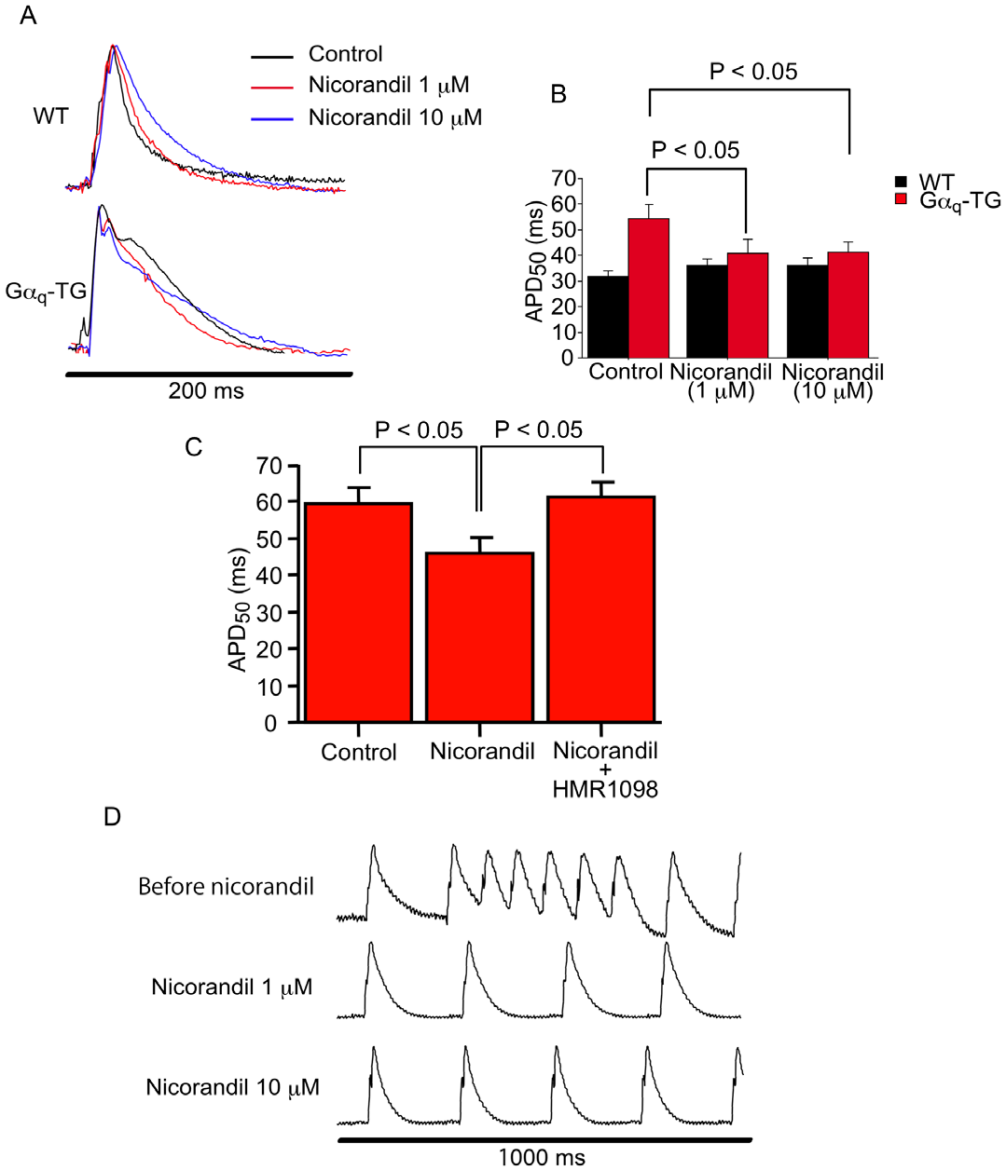
Effects of nicorandil on the ventricular action potential duration and spontaneous premature ventricular beats. Panel A: Representative examples of monophasic action potentials (MAPs) recorded from the posterior left ventricle in a Langendorff-perfused WT and Gα_q_-TG heart during steady state pacing at a cycle length of 200 msec. Panel B: Overall data for MAP duration in WT and Gα_q_-TG hearts before and after acute nicorandil administration. Panel C: Overall data for MAP duration in Gα_q_-TG hearts during the control, in the presence of nicorandil (10 µM) alone, and in the presence of nicorandil plus HMR1098 (30 µM), a blocker of cardiac sarcolemmal K_ATP_ channels. Panel D: Spontaneous premature ventricular beats in a Langendorff-perfused Gα_q_-TG heart before and after acute nicorandil administration. Mice at the age of 32 weeks were used. See text for details.

Shown in Figure 7A are representative examples of ventricular MAPs recorded from the posterior left ventricle in Langendorff-perfused WT and Gα_q_-TG hearts at the age of 32 weeks during steady state pacing at a cycle length of 200 msec. Ventricular MAP duration was prolonged in the Gα_q_-TG heart compared with the WT heart before acute nicorandil administration. After the nicorandil treatment, MAP duration shortened in the Gα_q_-TG heart but not WT heart (Fig. 7A). In addition, from the pooled data (Fig. 7B), nicorandil (1 and 10 µM) significantly shortened ventricular MAP duration in Gα_q_-TG hearts but not in WT hearts. Moreover, HMR1098 significantly attenuated the shortening of MAP duration induced by nicorandil in the Gα_q_-TG heart (Fig. 7C). Shown in Figure 7D are representative examples of ventricular repetitive beats in the Langendorff-perfued Gα_q_-TG heart during spontaneous sinus rhythm before and after acute nicorandil administration. PVC and ventricular repetitive beats were frequently observed in the Gα_q_-TG heart before nicorandil was administered (Fig. 7D, top). After the nicorandil treatment, MAP duration was shortened, and PVC and repetitive ventricular beats were not induced in the Gα_q_-TG heart (Fig. 7D, middle and bottom). High PVC was observed in 6 of 8 Langendorff-perfused Gα_q_-TG hearts before nicorandil treatment but only 1 of 8 Gα_q_-TG hearts after (p<0.05).

### Effects of Long-term Treatment with Nicorandil on WT Mouse Hearts

**Figure 8:**
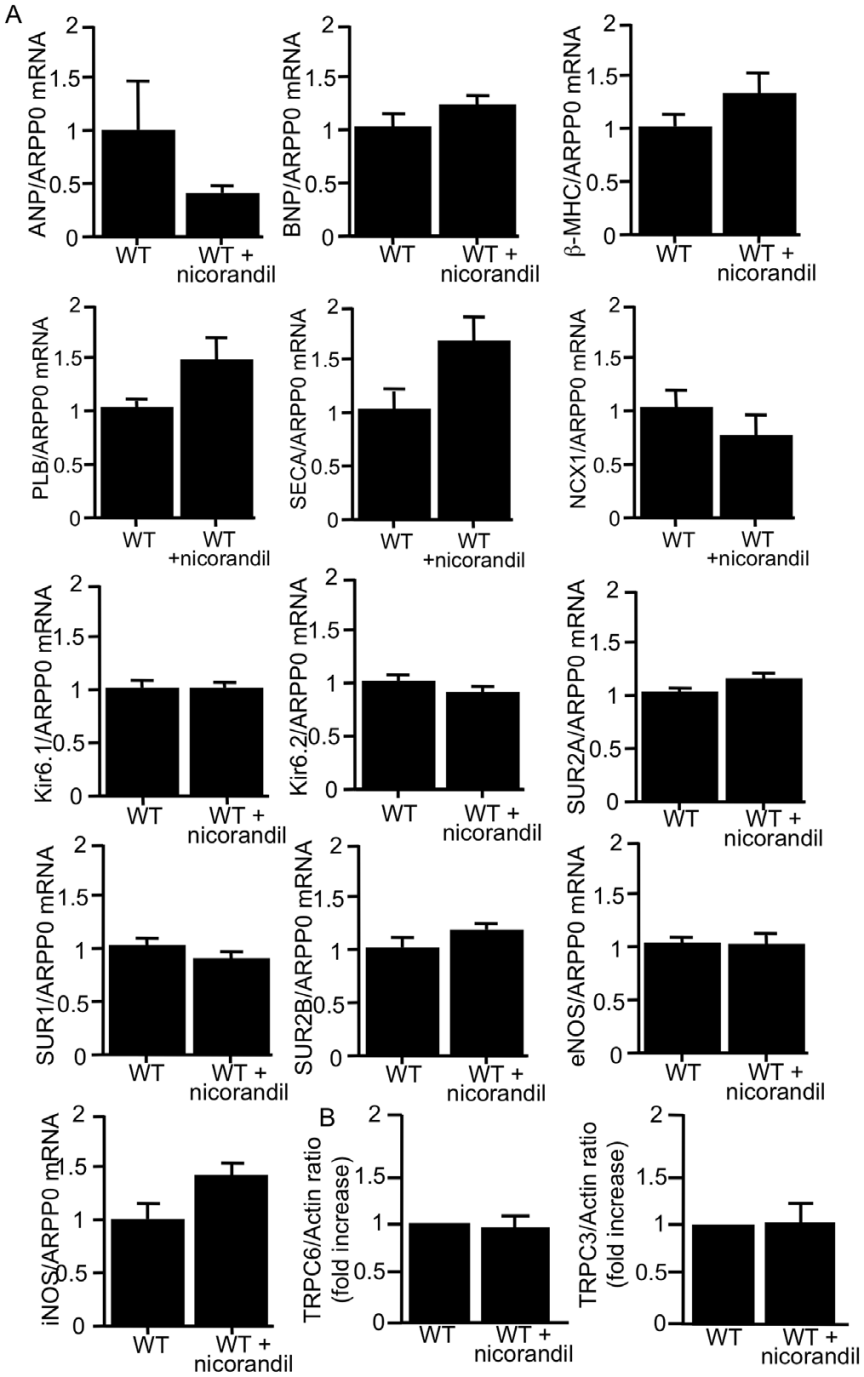
Effects of nicorandil on several gene expressions and on protein expression of TRPC channel isoforms in WT mice. Panel A: Quantitative analyses of ANP, BNP, β-MHC, PLB, SERCA2, NCX1, Kir6.1, Kir6.2, SUR1, SUR2A, SUR2B, eNOS, and iNOS gene expression by real-time RT-PCR in vehicle-treated WT (WT) and nicorandil-treated WT (WT+nicorandil) mouse hearts. The data were normalized to those for ARPP0. Data are the mean ± SE obtained from 5 mice for each group. Panel B: Expression of TRPC channel isoforms in WT+vehicle and WT+nicorandil hearts. TRPC isoform expression was normalized to actin expression and expressed relative to wt (set at 1). Data are the mean ± SE obtained from 5 mice for each group. Mice at the age of 32 weeks were used.

Chronic nicorandil treatment from 8 to 32 weeks of age had no effect on electrical and contractile function, hypertrophy, fibrosis, (Figs. 1, 2, 6, and Tables 1, 2, and 3). Figure 8 shows overall data for several genes and TRPC protein expression in WT and nicorandil-treated WT hearts. The chronic nicorandil treatment had no effect on gene and protein expression in WT mouse hearts (Fig. 8).

## Discussion

In this study, we found that chronic administration of nicorandil for 24 weeks prevented the progression of heart failure and ventricular arrhythmia in Gα_q_-TG mice. We also found that nicorandil inhibited ventricular interstitial fibrosis, attenuated the decreased SUR2B gene expression, and attenuated or prevented the decrease of eNOS and SERCA2 in Gα_q_-TG mouse hearts. It has been suggested that abnormalities in coronary hemodynamics in systolic heart failure contribute to ventricular remodeling, myocardial dysfunction and progressive heart failure. Both endothelium-dependent and -independent mechanisms of coronary vasodilatation are impaired in heart failure [18]–[20]. For example, intracoronary infusion of acetylcholine normally dilates the conductance and resistance vessels, and this vasodilatation is mediated by the release of endothelium-dependent relaxing factors/nitric oxide (NO). In patients with heart failure, intracoronary infusion of acetylcholine increases coronary artery resistance and decreases coronary blood flow [20]. Therefore, the decreased coronary blood flow reserve with a concurrent increase in myocardial oxygen demand might induce myocardial ischemia, myocyte necrosis and apoptosis in heart failure. Nicorandil is a K_ATP_ channel opener and a nitric oxide donor and widely used as a coronary vasodilator. K_ATP_ channels are comprised of a pore-forming subunit (Kir6.1 or Kir6.2) and a regulatory subunit, sulfonylurea receptors (SUR1 or SUR2). K_ATP_ channels with different combinations of these subunits exist in various tissues and regulate cellular functions, but the combination of Kir6.1 and SUR2B is mainly present in vascular smooth muscle including coronary artery [21]. Nicorandil more selectively activates K_ATP_ channels composed of Kir6.1 and SUR2B. Animal studies have shown that the opening of K_ATP_ channels increases endothelium-derived NO production by eNOS [17], inhibits endothelial cell death [22], and exerts anti-inflammatory [23] and antioxidative effects [24]. These findings suggest that improved endothelial function, anti-inflammatory effects, and reduced oxidative stress may all contribute to the long-term improvement of cardiovascular outcomes seen with nicorandil therapy. In this study, SUR2B gene expression was improved in nicorandil-treated Gα_q_-TG mouse hearts. Moreover, Nicorandil attenuated or prevented the decrease of eNOS in Gα_q_-TG hearts, suggesting that nicorandil improves coronary vasodilatation in this model. Nicorandil may also contribute to a potential upregulation of VEGF expression because deficiency of eNOS resulted in marked impairment of myocardial capillary development and the associated reduction in VEGF expression in the neonatal mouse myocardium [25]. These effects may also contribute to nicorandil-induced improvement of the coronary circulation. Nicorandil prevented decreases in SERCA2 in Gα_q_-TG mouse hearts. It is well known that decreased SERCA2 gene and protein expression causes an abnormal contractile function in failing hearts. Moreover, Koitabashi et al. [26] have suggested that carvedilol restores SERCA2 gene expression through prevention of oxidative stress-mediated downregulation of SERCA2 gene transcription, leading to the improvement of cardiac contractile function in failing hearts. Therefore, nicorandil may improve cardiac contractile function through the normalization of SERCA2 gene expression. In this study, nicorandil failed to improve the increased mRNA expression of profibrotic genes such as CTGF and collagen type 1, suggesting that nicorandil has no effect on the production of those proteins, although whether such protein expression in Gα_q_-TG mice increases or not is still unknown. Nevertheless, nicorandil administration inhibited the increased ventricular interstitial fibrosis. Nicorandil improved the decreased SUR2B and eNOS gene expression in Gα_q_-TG mouse hearts, which may contribute to the inhibition of the ventricular interstitial fibrosis because a previous study has demonstrated that nicorandil inhibits cardiac fibroblast proliferation induced by angiotensin II by activating K_ATP_ channels and the eNOS-NO pathway [27]. Therefore, nicorandil-induced coronary vasodilatation, upregulation of VEGF expression, improvement of SERCA2 gene expression, and inhibition of ventricular interstitial fibrosis might prevent the progression of heart failure. NO donors are commonly used in the management of patients with HF. However, the relevant clinical data do not manifest the broad beneficial effects observed in the present study with nicorandil. Moreover, a previous study has demonstrated that SUR2B immunoreactivity mainly occurred in the mitochondria as well as in the endoplasmic reticulum and cell membrane [28]. Thus, in this transgenic model of HF, the activation of K_ATP_ channels including that in the mitochondria may play an important mechanistic role in the beneficiary effects of nicorandil. In this study, we found that chronic nicorandil administration shortened the prolonged QT interval and reduced ventricular arrhythmias such as PVC in Gα_q_-TG mice. Numerous studies have characterized the ionic and molecular remodeling that occurs in the failing heart. One major change in the failing heart is a prolonged action potential duration which is linked mainly to downregulation of repolarizing potassium currents and an increase in late sodium current density [29]. HF-induced prolongation of action potential predisposes one to I_Ca-L_ reactivation underlying the generation of early after depolarization (EAD), which can act as initiating triggers for ventricular arrhythmias in HF. These results suggest that prevention of the progression of HF by chronic nicorandil treatment helps to prevent ventricular arrhythmias by shortening the prolonged QT interval in this model.

In this study, we found that acute nicorandil administration shortened ventricular monophasic action potential duration in Langendorff-perfused Gα_q_-TG hearts and reduced the number of PVCs. As described above, HF-induced action potential prolongation predisposes individuals to I_Ca-L_ reactivation underlying the generation of EAD, which can act as initiating triggers for ventricular arrhythmias in HF. In our previous study, EAD-induced triggered activity was frequently observed in single ventricular myocytes of Gα_q_-TG mice [11]. These results suggest that effects of acute nicorandil administration on ventricular action potential duration contributes to the inhibition of EAD-induced triggered activity and PVCs in Gα_q_-TG mice. Numerous studies have demonstrated that the pore-forming Kir6.2 and regulatory SUR2A subunits are essential elements of the sarcolemmal K_ATP_ channel in ventricular cardiomyocytes of several animals including mice. In this study, Kir6.2 and SUR2A gene expression was decreased in vehicle-treated Gα_q_-TG mouse hearts, and nicorandil did not prevent the decreased expression of these genes. Moreover, nicorandil selectively activates K_ATP_ channels composed of Kir6.1 and SUR2B. Nevertheless, nicorandil shortened the prolonged QT interval and ventricular action potential duration in Gα_q_-TG mouse hearts. In our previous study, we have shown that nicorandil dose-dependently shortened ventricular action potential duration before ischemia and augmented action potential duration shortening without increasing the dispersion of action potential duration during ischemia [6]. These effects of nicorandil were inhibited by HMR1098, suggesting that the activation of sarcolemmal K_ATP_ channels on the ventricular myocytes by nicorandil shortened APD homogenously. Moreover, our previous result suggested that nicorandil activates sarcolemmal K_ATP_ channels in cardiac muscle more effectively in ischemia than non-ischemia [30]. In fact, the present results demonstrated that HMR1098 significantly attenuated the shortening of MAP duration induced by nicorandil in the Gα_q_-TG heart. Therefore, nicorandil may more effectively shorten the prolonged ventricular action potentials in failing hearts in which relative myocardial ischemia might occur. Thus, nicorandil may prevent ventricular arrhythmias not only by preventing progressive HF but also by shortening action potential duration.

Several studies have shown that K_ATP_ channel agonists inhibit the development of cardiac hypertrophy. In fact, nicorandil inhibited cardiac hypertrophy after myocardial infarction through the inhibition of p70S6 kinase [31]. Moreover, a K_ATP_ channel agonist also inhibited the increased cell size in rat heart-derived H9c2 cells [32]. However, chronic administration of nicorandil for 24 weeks failed to inhibit the development of cardiac hypertrophy in Gα_q_-TG mice. It is well known that the development of cardiac hypertrophy is a multigenic, integrative response involving multiple pathways. A recent study has shown that activation of TRPC3 and 6 channels participates in the generation of cardiac hypertrophy [16]. Our previous study demonstrated that the protein expression of TRPC3 and 6 was increased in Gα_q_-TG mouse hearts [11]. Moreover, nicorandil did not inhibit the increased expression of TRPC 3 and 6 proteins and of fetal type genes in Gα_q_-TG hearts (Fig. 3). Therefore, the failure to completely abrogate the hypertrophic process was not surprising in view of the underlying complexity of the process.

While vehicle-treated Gα_q_-TG mice age-dependently exhibited reduced left ventricular contractility and chamber dilation between 8 and 32 weeks of age, nicorandil-treated Gα_q_-TG mice did not. Moreover, a previous study demonstrated that in Gα_q_-TG mice LVFS was decreased by approximately 27% at the age of 16 weeks similar to that at the age of 32 weeks in this study [10]. These results suggest that nicorandil prevents age-dependent progression of cardiomegaly and contractile dysfunction in Gα_q_-TG mice. Thus, the increased expression of eNOS, SUR2B, and SERCA2 mRNA may play important roles in prevention of the progression of heart failure in younger Gα_q_-TG mice.

## Supporting Information

Table S1 **Primers used in this study.**
(XLS)
